# Supplementary figures and images for: Bevacizumab-Induced Thrombotic Microangiopathy (TMA) in Metastatic Lung Adenocarcinoma Patients Receiving Nivolumab Combined with Bevacizumab, Carboplatin and Paclitaxel: Two Case Reports
Source: Clin Pract. 2023 Jan 30;13(1):200–5. doi: 10.3390/clinpract13010018 (PMC9955069; doi:10.3390/clinpract13010018)

Figure S1. Kidney ultrasonography of 2 study patients. (A) Patient #1 (B) Patient #2.

A.

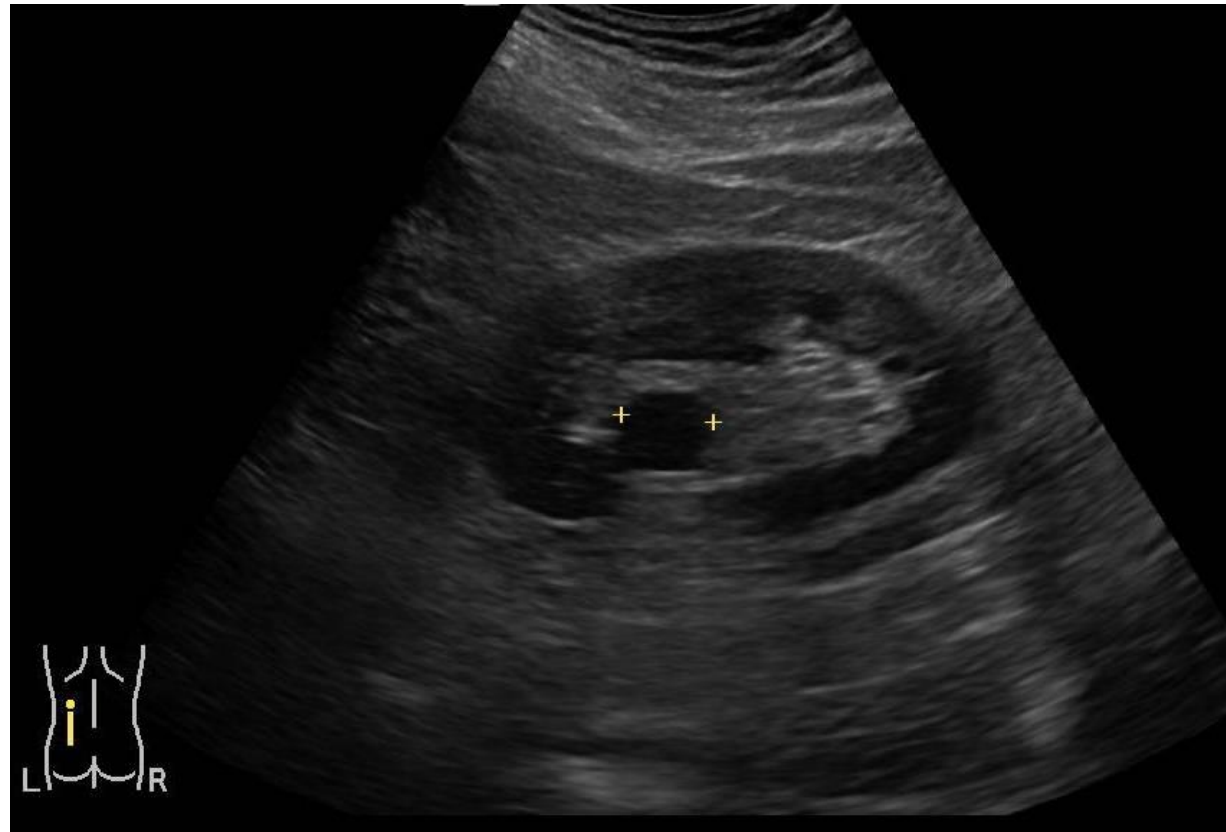

B.

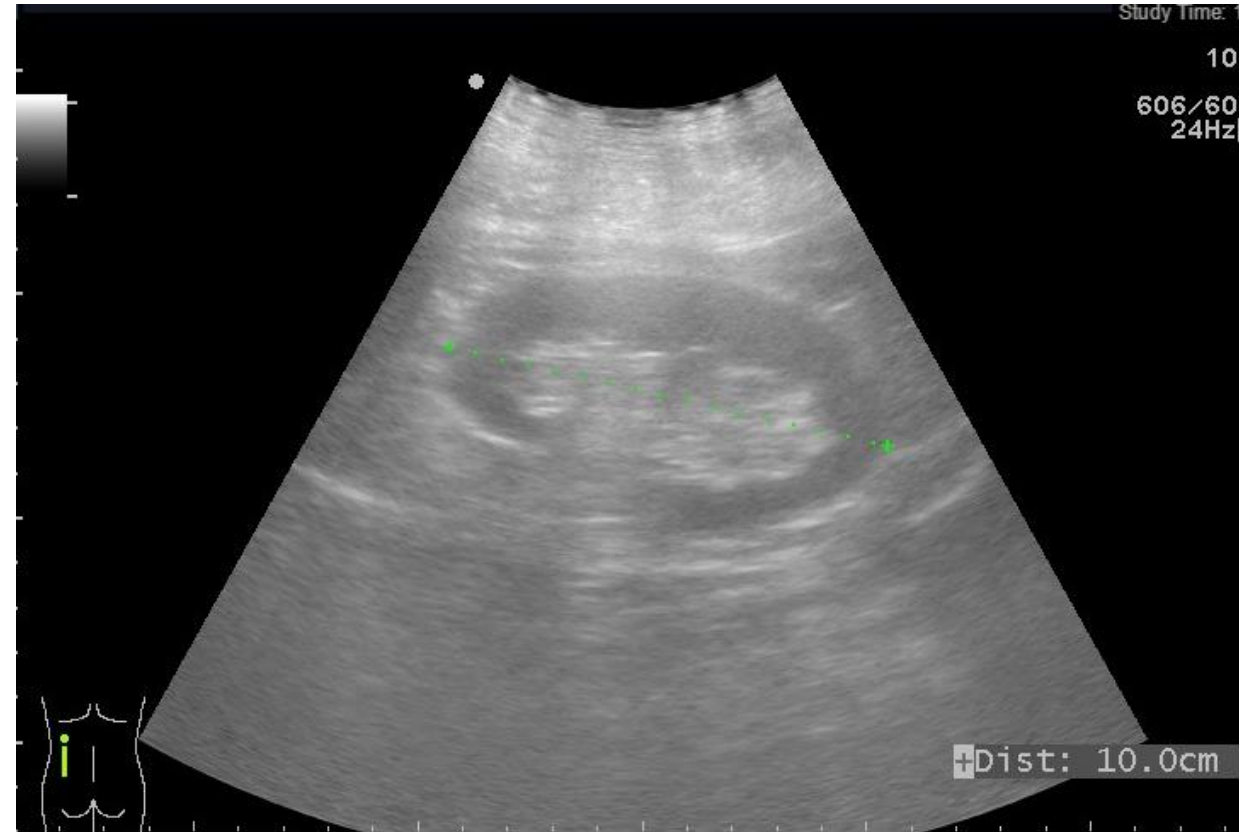

Supplement: Supplementary file 1 [file clinpract-13-00018-s001.zip › clinpract-2097717-supplementary.pdf]
